# Salivary Microbiome Profile of Diabetes and Periodontitis in a Chinese Population

**DOI:** 10.3389/fcimb.2022.933833

**Published:** 2022-08-01

**Authors:** Chunting Lu, Qingtong Zhao, Jianwen Deng, Kexiao Chen, Xinrong Jiang, Fengyu Ma, Shuyuan Ma, Zejian Li

**Affiliations:** ^1^ Science and Education Office, The First Affiliated Hospital, Jinan University, Guangzhou, China; ^2^ Department of Stomatology, The Sixth Affiliated Hospital of Jinan University, Dongguan, China; ^3^ School of Stomatology, Jinan University, Guangzhou, China; ^4^ Medical Center of Stomatology, The First Affiliated Hospital, Jinan University, Guangzhou, China; ^5^ Chaoshan Hospital, The First Affiliated Hospital of Jinan University, Chaozhou City, China

**Keywords:** diabetes, periodontitis, saliva, 16S rDNA, microbiota

## Abstract

**Aim:**

There is a bidirectional association between diabetes and periodontitis. However, the effect of diabetes on the periodontitis salivary microbiota has not been elucidated. The aim of this study was to determine the effect of the presence of diabetes on the microbiota among Chinese patients with periodontitis.

**Materials and Methods:**

Unstimulated whole saliva samples were collected from the periodontitis with diabetes group (TC), chronic periodontitis group (CP), and periodontally healthy and systemically healthy group (H) by spitting method. Bacterial genomic DNA was PCR-amplified at the V4 variable region of 16S rRNA gene. The library was constructed according to the obtained sequence results, and biological analysis and statistical analysis were carried out. Functional prediction of three groups of microbial communities was performed by the PICRUSt algorithm.

**Results:**

There was no significant difference in bacterial diversity between the TC and CP groups. Compared with the H group, the TC group and CP group presented a higher diversity of salivary flora. *Firmicutes*, *Streptococcus*, *Haemophilus*, *Veillonella*, and *Haemophilus parainfluenzae* dominated the H group. *Corynebacterium*, *Leptotrichia*, *Dialister*, *Comamonas*, *Capnocytophaga*, *Catonella*, *Filifactor*, *Campylobacter*, *Treponema*, *Campylobacter concisus*, *Prevotella oralis*, and *Porphyromonas gingivalis* were significantly enriched in the TC and CP groups. Among them, *Treponema* and *P. oralis* were the most abundant in the TC group. The PICRUSt results showed that many pathways related to cell motility and functional metabolism of the salivary microbial flora changed in the TC group and the CP group.

**Conclusions:**

Diabetes was not the main factor causing the altered diversity of salivary microbiota in patients with periodontitis; however, the presence of diabetes altered the abundance of some microbiota in saliva.

## Introduction

Diabetes mellitus is a common metabolic disorder characterized by chronic hyperglycemia. The latest epidemiological studies have shown that more than 440 million people worldwide suffer from diabetes (Lovic et al., 2020), mainly in countries in the Asia-Pacific region. According to statistics, China has the largest number of diabetic patients in the world (Ma, 2018). Therefore, diabetes is an unresolved public health problem in China and throughout the world.

Periodontitis is the sixth most common complication in people with diabetes (Löe, 1993). Current epidemiological investigations have demonstrated a bidirectional relationship between diabetes and periodontitis. Type 2 diabetes mellitus (T2DM) is the most common type of diabetes, and its age of onset is more similar to periodontitis. Considering the different etiologies of type 1 diabetes (T1DM) and T2DM, and the potential differences in the pathobiology of these two diseases, it is more beneficial to explore their effects on periodontitis separately to explain their relationship with periodontitis. Systemic effects associated with T2DM and periodontitis may act synergistically to produce more severe periodontal disease progression than T1DM (Novak et al., 2008). Increased severity of periodontal disease in T2DM may reflect the altered pathogenic potential of periodontal bacteria and/or altered host inflammatory response characteristics, which may lead to a breakdown of periodontal homeostasis (Naguib et al., 2004). However, studies on the effect of T2DM on the periodontitis microbiota have yielded conflicting results (Polak and Shapira, 2018). This may be due to factors such as different regions, ethnic differences, and age (Taylor et al., 2013). Therefore, it is necessary to conduct in-depth research on the differences in the microbiota structure of periodontitis patients with or without diabetes among different populations.

In the Chinese population, the impact of diabetes on periodontitis salivary flora is relatively unknown. In the study of inflammatory mediators and microbiota in periodontitis and systemic diseases, saliva has been proven to be a viable substitute for serum and gingival crevicular fluid (Deepa and Thirrunavukkarasu, 2010), which helps to solve some problems inherent to gingival crevicular fluid sampling (Ghallab, 2018), such as time consumption, multiple sampling, and blood contamination. With the loss of periodontal attachment and the appearance of deep periodontal pockets, many serum-like fluids and microorganisms in gingival crevicular fluid infiltrate into saliva; thus, most periodontal pathogens and inflammatory mediators can be detected in saliva (Kim et al., 2018). Therefore, this study used a Chinese population as the research object to explore the impact of diabetes on periodontitis-related microorganisms in the saliva, in order to provide a theoretical basis for further research on the microbial mechanism of diabetes on periodontitis.

## Methods

### Study Population

The subjects of the study were patients who visited the Department of Stomatology and Endocrinology of the First Affiliated Hospital of Jinan University from January 2021 to January 2022. Thirty volunteers were recruited by convenience sampling and divided into the periodontitis with diabetes group (TC group, n = 10), chronic periodontitis group (CP group, n = 10), and healthy group (H group, n = 10).

Periodontitis was defined by the presence of alveolar bone resorption in at least 30% of sites and more than 4 sites with probing depth (PD) ≥4 mm and clinical attachment loss (CAL) ≥2 mm (Zhou et al., 2013). T2DM was defined as glycated hemoglobin (HbA_1_C) ≥6.5% and fasting blood glucose (FBG) ≥7.0 mmol/L (Maboudi et al., 2019). Individuals who were diagnosed with periodontitis and had no history of diabetes, self-reported systemic health, or blood tests with normal HbA_1_C were included in the CP group. Patients newly diagnosed with T2DM in the endocrinology department and periodontitis diagnosed in the stomatology department were included in the TC group. Individuals without periodontitis and self-reported systemic health were included in the H group.

The following patients were excluded from our study: 1) any patient diagnosed with metabolic disease other than diabetes and systemic disease that may have affected the progression of the periodontitis, such as hyperthyroidism or cancer; 2) prior to the experimental history of special medication, such as antibiotics or hormones, within 6 months; 3) received periodontal treatment, such as scaling, within 6 months; 4) pregnant or breastfeeding; 5) current smokers (Becker et al., 2014); 6) remaining tooth with root caries. All participants signed a written informed consent form and agreed to use their own saliva samples for this study. This study was approved by the Medical Ethics Committee of the First Affiliated Hospital of Jinan University in China (Approval Number Kyk-2022-014) and strictly followed the Declaration of Helsinki.

### Saliva Collection and DNA Extraction

Before the periodontal assessment, unstimulated whole saliva samples were collected by spitting. All research subjects were asked to fast for at least 8 h and to avoid eating, rinsing, brushing their teeth, and performing other oral exercises for at least 2 h before sampling. Then, 2 ml of saliva was collected between 9:00 am and 11:00 am and immediately stored in a −80°C refrigerator. The genomic DNA of the samples was extracted by the conventional cetyl trimethylammonium bromide method (CTAB), and the purity and concentration of the DNA were then detected by agarose gel electrophoresis.

### High-Throughput Sequencing

With the use of the diluted genomic DNA as a template and specific primers with Barcode, PCR amplification was performed on the 16S rRNA gene V4 variable region. The forward primer sequence was 515F (5′-GTGCCAGCMGCCGCGCTAA-3′), and the reverse primer sequence was 806R (5′-GGACTACHVGGGTTWTCTAAT-3′). The TruSeq^®^ DNA PCR-Free Sample Preparation Kit (Illumina, San Diego, CA, USA) was used for library construction. The library quality was assessed on the Qubit@ 2.0 Fluorometer (Thermo Scientific, Waltham, MA, USA) and Agilent Bioanalyzer 2100 system (Agilent Technologies, Santa Clara, CA, USA). After the library was qualified, the Illumina NovaSeq 6000 platform was used for on-machine sequencing.

### Biological Analysis and Statistical Analysis

The original sequence was spliced and filtered to obtain clean data (effective tags). Then, Uparse software (Uparse v7.0.1001, http://www.drive5.com/uparse/) was used to perform operational taxonomic unit (OTU) clustering on the effective tags of all samples. Species annotation taxonomic analysis was performed using the Silva database (http://www.arb-silva.de/) based on the Mothur algorithm. Differences in dominant species between groups were compared using MUSCLE software (version 3.8.31, http://www.drive5.com/muscle/). After the data were normalized, alpha diversity analysis and beta diversity analysis were performed using QIIME software (Version 1.9.1) and R software (Version 2.15.3). With the use of R software (Version 2.15.3), statistical analysis methods, such as T-test, MetaStat, and analysis of similarity (ANOSIM), were used to test the significance of differences in the species composition and community structure of the grouped samples. PICRUSt predicted microbial function, and a T-test was used to detect functional differences between groups. Statistical analysis was performed on the baseline data of the study population using ANOVA or Tukey’s test and the chi-square test in GraphPad Prism software.

## Results

### Sociodemographic Characteristics and Clinical Examination

As shown in **Table 1**, there was no significant difference in age or sex among the TC group, CP group, and H group (p > 0.05). There were significant differences in CAL and PD between the CP group, TC group, and H group (p < 0.05), but there were no significant differences in CAL or PD between the CP group and TC group (p > 0.05). The average levels of HbA_1_C and FBG in the TC group were 8.54% ± 1.38% and 12.03 ± 2.63 mmol/L.

**Table 1:** Social demographic characteristics and clinical parameters among TC, CP, and H groups.

Variable	TC	CP	H	P-value
	(mean ± SD)	(mean ± SD)	(mean ± SD)	
Age (years)	36.10 ± 2.60	32.40 ± 6.26	32.70 ± 3.56	p > 0.05^a^
Sex (male/female)	5/5	6/4	5/5	p > 0.05^b^
PD (mm)	4.51 ± 0.53^#c^	4.09 ± 0.38^#c^	1.65 ± 0.29	p < 0.05^a^
CAL (mm)	2.27 ± 0.98^#c^	1.78 ± 0.51^#c^	0.00 ± 0.00	p < 0.05^a^
HbA_1_C (%)	8.54 ± 1.38	–	–	–
FBG (mmol/L)	12.03 ± 2.63	–	–	–

PD, pocket depth; CAL, clinical attachment loss; HbA_1_C, glycosylated hemoglobin; FBG, fasting blood glucose. p < 0.05 is statistically significant.

^#^ p > 0.05 between TC and CP groups.

a ANOVA.

b Chi-square test.

c Turkey test.

### Operational Taxonomic Unit and Sequencing Depth Analysis

**Figure 1:**
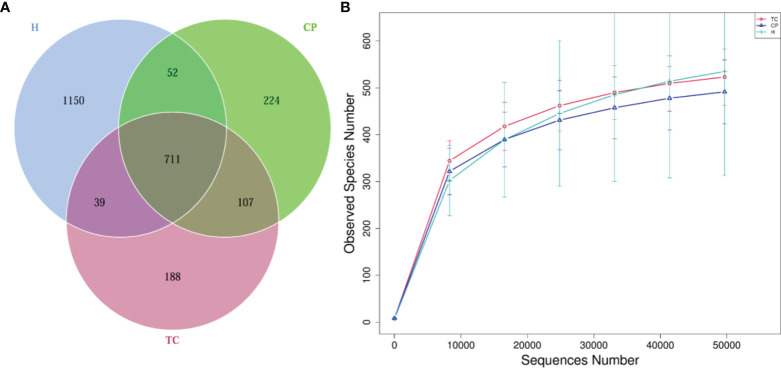
Venn diagram and rarefaction curve diagram. **(A)** Venn diagram. Each circle in the figure represents a group, the numbers in the overlapping parts of the circles and the circles represent the number of operational taxonomic units (OTUs) shared among the samples (groups), and the numbers without overlapping parts represent the unique OTUs of the groups. **(B)** Rarefaction curve diagram. The abscissa is the number of sequencing strips randomly selected from a sample, and the ordinate is the number of OTUs that can be constructed based on the number of sequencing strips. Different groups are represented by curves with different colors.

To study the bacterial community composition of each sample, OTUs were clustered with 97% consistency for the effective tags of all samples, and species annotation was then performed on the sequences of OTUs. In total, 2,471 OTUs were identified in this study, from which 22 phylum-, 277 genus-, and 264 species-level taxa were detected. As shown in **Figure 1A**, 711 OTUs overlapped in the three sets of samples. The CP group had 1,094 OTUs, the TC group had 1,045 OTUs, and the H group had 1,952 OTUs. The CP group had 224 unique OTUs, the TC group had 188 unique OTUs, and the H group had 1,150 unique OTUs. The sparsity curve among the three groups was flat (**Figure 1B**), indicating that the sequencing sampling of the study was reasonable and that more data volumes would yield only a small number of new species.

### Microbial Diversity Analysis

**Figure 2:**
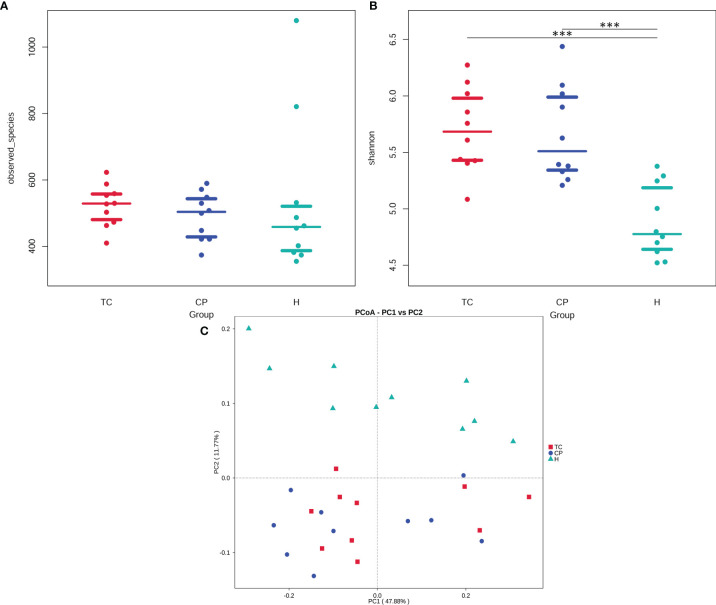
Diversity analysis plot. **(A)** Beeswarm of the observed species index. **(B)** Beeswarm of the Shannon index. **(C)** Principal coordinate analysis (PCoA) plot. The samples of the same group in the figure are represented by the same color, each point represents a sample, and the distance between the points represents the degree of difference.

The alpha diversity among the three groups is shown in **Figures 2A, B**. From the Beeswarm of the observed species (**Figure 2A**), there was no significant difference in species abundance among the three groups (p > 0.05). The Beeswarm of Shannon indicated (**Figure 2B**) that the diversity of the TC group and the CP group was significantly increased compared with that of the H group. However, there was no significant difference in species abundance or diversity between the TC group and the CP group.

The beta diversity among the three groups is shown in **Figure 2C**. The principal coordinate analysis (PCoA) based on the weighted UniFrac distance showed that the samples in the TC group and the CP group were closer in distance, indicating that the degree of bacterial evolution of the samples between the two groups was similar, and the bacterial community structure between the two groups was similar. The distance between the H group and the TC and CP groups was farther, indicating that the bacterial community structures of the disease study group (TC and CP groups) and the H group were significantly different. ANOSIM further showed that there was no significant difference in the flora structure between the TC group and the CP group (R^2^ = 0.008, p > 0.05). The bacterial community structure between the CP group and the H group (R^2^ = 0.162, p < 0.001) and the TC group and the H group (R^2^ = 0.204, p < 0.001) were statistically significant.

### Differences in Bacterial Community Structure Between Groups at the Phylum Level

**Figure 3:**
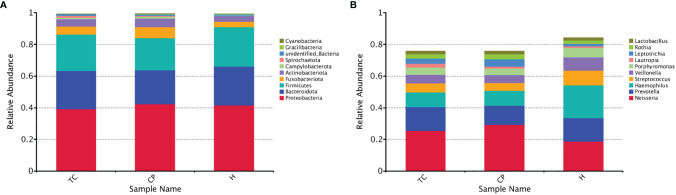
Species relative abundance column chart. **(A)** Phylum level. **(B)** Genus level.

**Figure 4:**
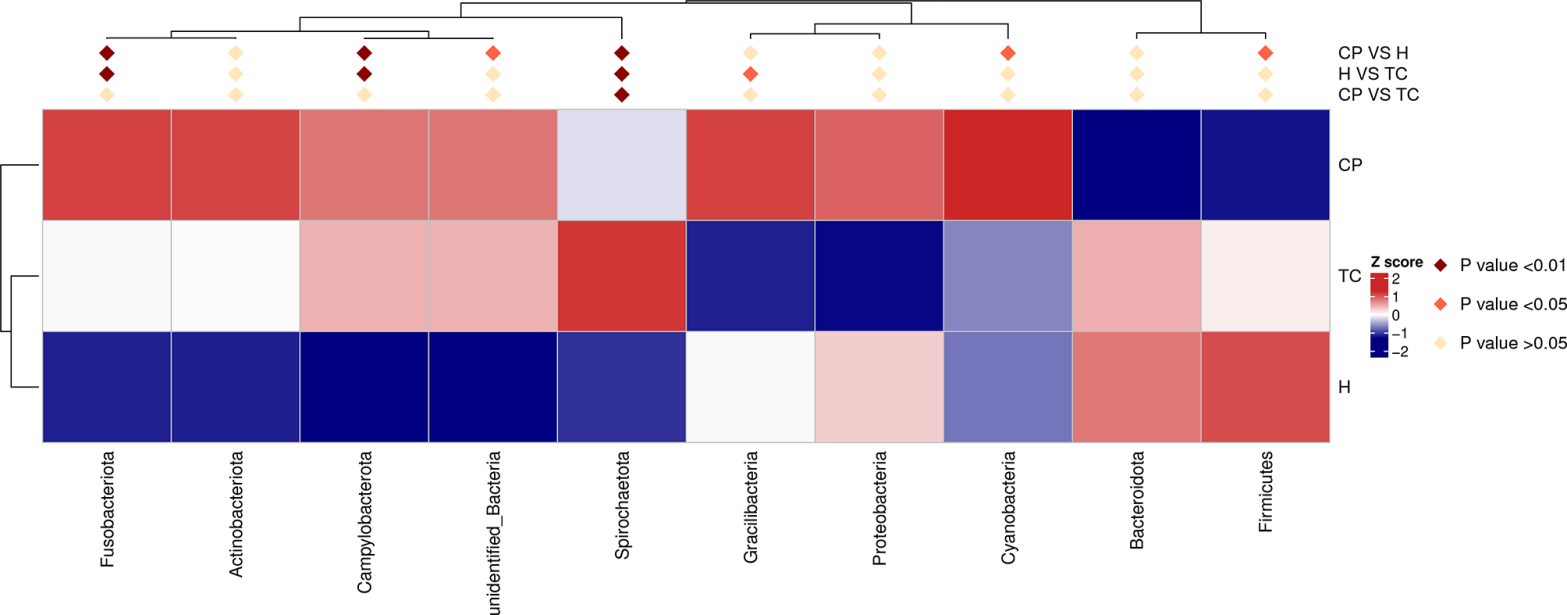
MetaStat analysis; phylum annotation heatmap. Different colors in the heatmap represent the values obtained after the Z score of the relative content of differential metabolites is standardized, reflecting the level of their relative content (red represents high content, and blue represents low content).

As shown in **Figure 3A**, at the phylum level, the three groups of samples were dominated by Proteobacteria, Bacteroidota, Firmicutes, Fusobacteriota, and Actinobacteria. The MetaStat and T-test results (**Figure 4** and **Supplementary Figure S1**) showed that the phyla Fusobacteriota, Cyanobacteria, and Spirochaetota in the TC group were higher than those in the H group, and the phylum Gracilibacteria was lower than that in the H group; the phyla Fusobacteriota, Campylobacterota, Spirochaetota, and Cyanobacteria in the CP group were higher than those in the H group, while Firmicutes was lower than that in the H group; Spirochaetota was higher in the TC group than in the CP group.

### Differences in Bacterial Community Structure Between Groups at the Genus Level

**Figure 5:**
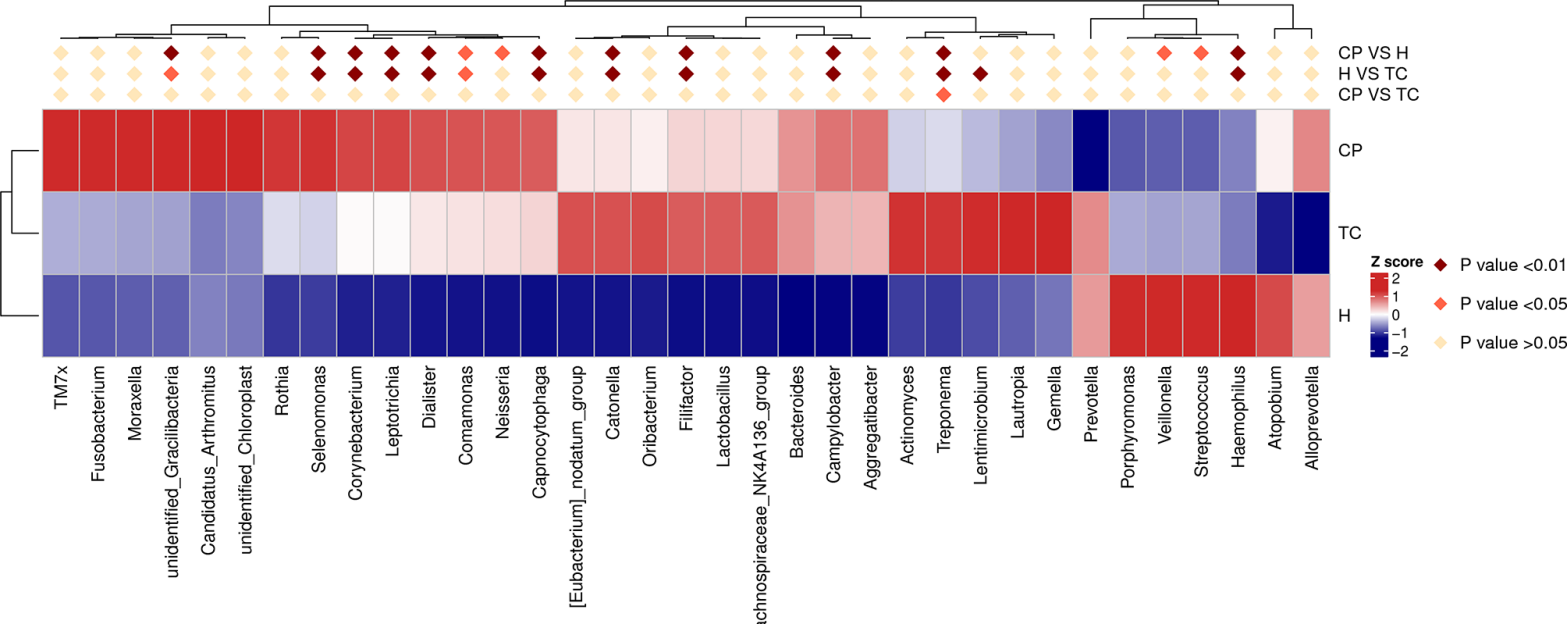
MetaStat analysis; genus annotation heatmap. Different colors in the heatmap represent the values obtained after the Z score of the relative content of differential metabolites is standardized, reflecting the level of their relative content (red represents high content, and blue represents low content).

As shown in **Figure 3B**, the genus-level distribution among the three groups of samples was in the order of *Neisseria*, *Prevotella*, *Haemophilus*, *Streptococcus*, *Veillonella*, and *Porphyromonas*. The MetaStat and T-test results (**Figure 5** and **Supplementary Figure S2**) showed that the *Leptotrichia*, *Selenomonas*, *Campylobacter*, *Catonella*, *Treponema*, *Corynebacterium*, *Capnocytophaga*, *Dialister*, *Comamonas*, and *Filifactor* genera were higher in the TC group than in the H group; *Haemophilus* was lower than that in the H group; the *Neisseria*, *Leptotrichia*, *Campylobacter*, *Catonella*, *Treponema*, *Corynebacterium*, *Capnocytophaga*, *Dialister*, and *Filifactor* genera were higher in the CP group than in the H group, and *Haemophilus*, *Streptococcus*, and *Veillonella* were lower than those in the H group; the *Treponema* genus was higher in the TC group than the CP group.

### Differences in Bacterial Community Structure Between Groups at the Species Level

**Figure 6:**
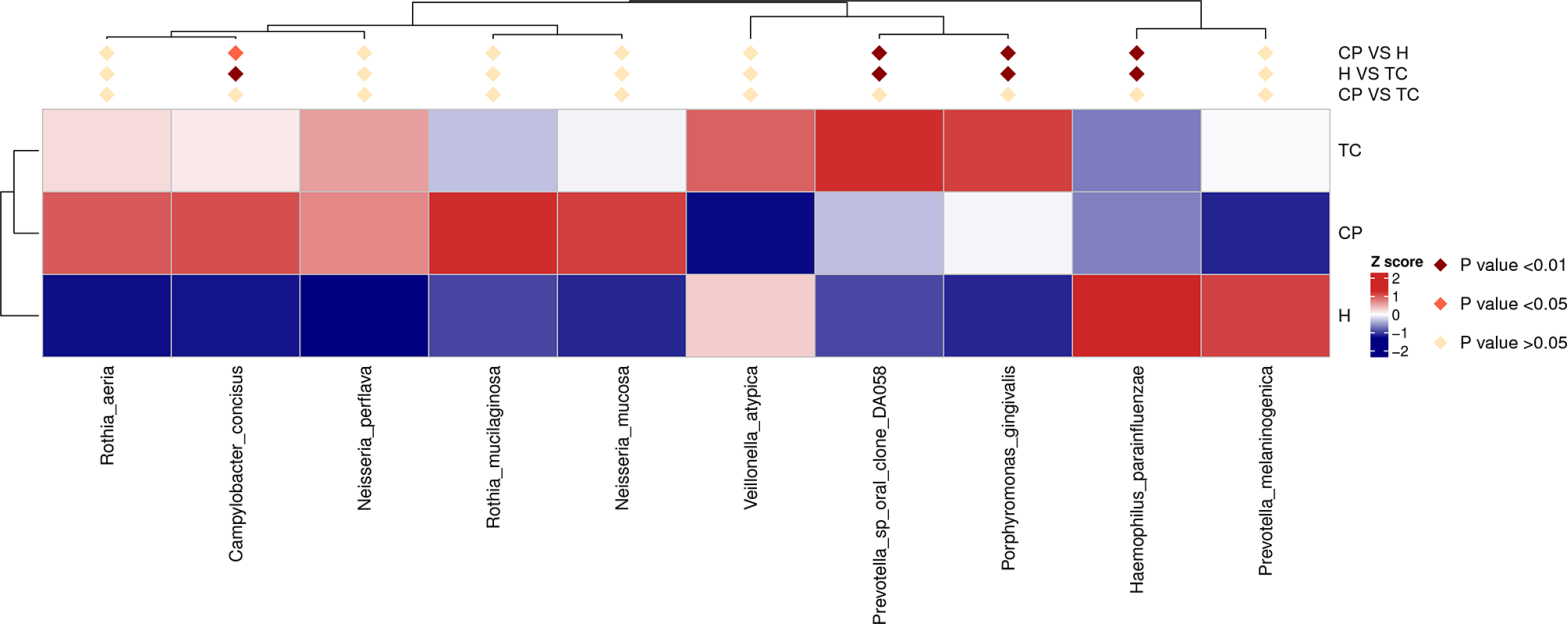
MetaStat analysis; species annotation heatmap. Different colors in the heatmap represent the values obtained after the Z score of the relative content of differential metabolites is standardized, reflecting the level of their relative content (red represents high content, and blue represents low content).

The MetaStat and T-test results (**Figure 6** and **Supplementary Figure S3**) showed that, at the species level, *Campylobacter concisus*, *Prevotella oris*, *Porphyromonas gingivalis*, and *Prevotella intermedia* were higher in the CP group and TC group than in the H group and that *Haemophilus parainfluenzae* was lower than that in the H group. The relative abundances of *P. intermedia* were higher in the TC group, compared with the CP group (**Supplementary Figure S3**).

### Predictive Analysis of Salivary Microbial Community Function

Using the PICRUSt algorithm, we performed functional predictions based on the Kyoto Encyclopedia of Genes and Genomes (KEGG) database for the three groups of microbial communities. As shown in **Supplementary Figures S4A, B**, at level 1, there were significant differences in the genetic information processing and the functions of cellular processes between the TC group, the CP group, and the H group (p < 0.05). As shown in **Supplementary Figures S4C, D**, **S5**, and **S6**, at level 2 and level 3, the TC group exhibited significantly enriched cellular processes (bacterial motility proteins, flagellar assembly, and bacterial chemotaxis), signaling (two-component system), basal energy (oxidative phosphorylation), and amino acid metabolism (aspartate metabolism, glutamate metabolism, histidine metabolism, tyrosine metabolism, tryptophan metabolism, etc.); the CP group showed significantly enriched amino acid metabolism (alanine, aspartic acid, glutamine, lysine, histidine, tyrosine, tryptophan, phenylalanine, etc.), cellular processes (cell motility, bacterial motility proteins, flagellar assembly, and bacterial chemotaxis), membrane transport (secretory system), basal energy (energy metabolism and oxidative phosphorylation), and other functions; group H showed significantly enriched replication and repair, translation, nucleotide metabolism (purine metabolism, pyrimidine metabolism) and other functions.

## Discussion

Periodontitis is an inflammatory disease that is thought to be associated with a variety of microbial disturbances (Darveau, 2010). Considering the bidirectional relationship between diabetes and periodontitis, in the context of diabetes, the bacterial community structure and diversity of periodontitis may be altered, thereby exacerbating periodontal bone loss. In recent years, many countries have carried out research on the impact of diabetes on periodontitis-related flora. However, the findings of these studies are not necessarily applicable to the Chinese population. Therefore, based on the Chinese population, we explored the influence of the presence or absence of diabetes on the salivary flora of periodontitis patients.

Similar to our findings, Liu (Liu et al., 2021) and Farina (Farina et al., 2019) et al. showed that diabetes did not affect the diversity of salivary microbiota in the context of periodontitis disease. Previous studies have shown that the presence of diabetes alone does not affect the structure of the salivary flora. This is further supported by studies by Almeida-Santos (Almeida-Santos et al., 2021) and Shi et al. (2020) In the study by Sun et al., periodontitis patients with and without T2DM had an increased diversity of salivary flora compared with healthy controls, while there was no significant difference between T2DM and periodontitis with T2DM patients after active blood sugar control (Sun et al., 2020). Therefore, they believe that periodontitis-related parameters are the main factors affecting the composition of salivary microbes, and the combined effect of T2DM and periodontitis on changes in the salivary microbiome is significantly greater than that of T2DM alone.

Compared with the H group or the CP group, the TC group showed an increasing trend in the flora biodiversity. This is similar to that reported by Balmasova (Balmasova et al., 2021) and Chen (Chen et al., 2020). However, Sabharwal (Sabharwal et al., 2019) and Saeb et al. (2019) believed that the presence of diabetes reduces the diversity of periodontitis salivary flora. These studies have different viewpoints, which may be attributed to the following reasons: 1) the sample size of each study was different, and small sample sizes may lead to a limited detection rate of flora related to the background of periodontitis. 2) Salivary flora may be affected by blood sugar status and different stages of periodontal disease, but many studies have not strictly distinguished whether the differential changes in salivary flora are caused by hyperglycemia or periodontal disease. 3) Studies have shown that the microbiota of stimulated saliva samples and unstimulated saliva samples is different (Gomar-Vercher et al., 2018); thus, the different ways of collecting saliva samples in each study may have affected the microbiota analysis results.

The dominant bacterial phyla in saliva were mainly Firmicutes, Bacteroidota, Proteobacteria, Actinobacteria, and Fusobacteriota (Lazarevic et al., 2010; Wade, 2013), among which Firmicutes were mainly enriched in healthy people (Griffen et al., 2012; Esparbès et al., 2021). This is largely consistent with the findings of this study. The statistical analysis of the differences in community structure at the phylum level showed that the common dominant bacterial phyla in the TC and CP groups were Fusobacteriota, Spirochaetota, and Campylobacterota, and Spirochaetota was more significantly enriched in the TC group. The Spirochaetota phylum is mainly composed of the genus *Treponema*. At the genus level, differential expression of this genus was observed in the TC group. It has been reported that the Spirochaetota flora accounts for 50% of the subgingival microbial population in periodontitis and less than 1% in healthy individuals (Chan and McLaughlin, 2000). They were found to be significantly higher in relative abundance in moderate-to-severe periodontitis (Listgarten and Levin, 1981; Armitage et al., 1982). The results of Marotz et al. confirmed that the log ratio of *Treponema* and *Corynebacterium* is a novel microbial indicator of periodontitis, and the increase in the detection rate of *Treponema* predicts the trend of periodontitis (Marotz et al., 2022). Diabetes has been reported to significantly increase the expression levels of these genera in the oral cavity (Sabharwal et al., 2019; Sun et al., 2020; Balmasova et al., 2021). A potential reason for the selective increase in this microbiota in periodontitis with diabetes may be the altered microenvironment due to differences in blood glucose, leading to the preferential proliferation of microorganisms with different growth requirements in specific oral niches.

At the genus level, the three groups of saliva samples were dominated by *Neisseria*, *Prevotella*, and *Haemophilus* species. The relative relationship between *Corynebacterium*, *Leptotrichia*, *Dialister*, *Comamonas*, *Capnocytophaga*, *Catonella*, *Filifactor*, *Campylobacter*, and *Treponema* in the TC and CP groups was higher than that in the H group, while *Haemophilus*, *Veillonella*, and *Streptococcus* were more abundant in the H group. In the process of plaque formation, *Streptococcus*, *Haemophilus*, *Veillonella*, *Actinomyces*, and *Fusobacterium* are the main flora in the early stage of bacterial colonization (Marsh, 2010). Periodontal disease-associated flora was observed to increase in abundance within the plaque as the plaque matured (Borisy and Valm, 2021). Belstrøm et al. suggested that *Streptococcus* gradually decreased in the late stage of oral plaque formation, while the abundance of some periodontal disease-related genera, such as *Leptotrichia* and *Prevotella*, gradually increased (Belstrøm et al., 2018). Therefore, the differences in the composition of microflora in saliva may indirectly reflect different stages of plaque formation and different stages of periodontitis. At the genus level, oral *Streptococcus* was confirmed to be the most abundant genus in healthy populations. However, *Haemophilus*, *Veillonella*, *Prevotella*, *Leptotrichia*, *Campylobacter*, *Neisseria*, and *Capnocytophaga* were not only expressed in periodontitis but also enriched in the periodontally healthy (Cao et al., 2018; Chen et al., 2018; Abusleme et al., 2021). This may be due to differences in the pathogenic potential of bacteria of the same genus.

At the species level, the dominant floras of the TC group and CP group were *C. concisus*, *Prevotella oralis*, and *P. gingivalis*, while the dominant flora of the H group was *H. parainfluenzae*. The presence of diabetes increased the expression of *P. intermedia* in saliva. These two bacteria belong to a core group closely associated with periodontitis and were confirmed to be positively associated with the subgingival plaque flora (Sakamoto et al., 2000; Belstrøm et al., 2017). Therefore, we speculate that patients with T2DM are more susceptible to the dysbiosis of oral flora, which in turn increases the risk and severity of periodontitis. In fact, the role of *P. gingivalis* and *Prevotella* (e.g., *P. intermedia*) in periodontitis has been extensively studied, while research on *C. concisus* in periodontitis is still limited. Studies have reported that the abundance of *C. concisus* is associated with increased levels of attachment loss in subjects with periodontitis (Haffajee et al., 1984). Henne et al. suggested that periodontitis does not affect changes in the absolute abundance of periodontitis and does not affect absolute changes in the abundance of *C. concisus* in the oral cavity, but the proportion of different species within *Campylobacter* may reflect microbial community changes as periodontitis progresses (Henne et al., 2014). The role of *Campylobacter* in periodontitis still needs further research in the future.

This study used the PICRUSt algorithm to predict the biological functions of differential enrichment of salivary microbiota in different groups. It is worth noting that cell motility, bacterial motility proteins, flagellar assembly, and bacterial chemotaxis were significantly enriched in the TC and CP groups. These biological functions related to the microbial virulence system can promote the adhesion and invasion of pathogenic microorganisms in periodontal tissue and then play an important role in the pathological process of periodontitis (Cai et al., 2021). In addition, similar to previous studies (Ikeda et al., 2020), we observed upregulation of pathways related to the metabolism of amino acids, such as aspartate, glutamate, tyrosine, tryptophan, and phenylalanine, in the TC group and the CP group. Although the underlying mechanism of amino acids in diabetes and periodontitis is not clear, amino acid metabolism (e.g., phenylalanine, tryptophan, and tyrosine) has been associated with the risk of diabetes and periodontitis in previous studies (Chen et al., 2016; Liebsch et al., 2019). Increases in certain amino acid catabolism-derived metabolites may induce disruption of host homeostasis, which in turn promotes the progression of periodontal inflammation (Kim, 2011).

It is worth noting that the small sample size of this study may have limited the detection rate of microbiota. In addition, we did not consider the effect of different periodontitis states on the expression of salivary flora. Therefore, the findings of this study need to be interpreted with caution. Nonetheless, we detected differential expression of some microbiota in periodontitis saliva in the presence of diabetes, and these results may inform future studies of these two common diseases.

## Conclusions

Diabetes was not the main factor causing the altered diversity of salivary microbiota in patients with periodontitis, but the presence of diabetes altered the expression abundance of some microbiota in saliva.

## Data Availability Statement

The datasets presented in this study can be found in online repositories. The name of the repository and accession number can be found below: NCBI; PRJNA843376.

## Ethics Statement

The studies involving human participants were reviewed and approved by The Ethics Committee of the First Affiliated Hospital of Jinan University, China. The patients/participants provided their written informed consent to participate in this study.

## Author Contributions

ZL designed the research project. CL, QZ and JD conducted experiments and contributed significantly to analysis and manuscript preparation. KC and XJ performed the data analyses and wrote the manuscript. FM and SM helped perform the analysis with constructive discussions. All the authors discussed and analyzed the experimental data, and wrote and revised the manuscript. All authors have reviewed the manuscript.

## Funding

This work was partly supported by the National Nature Science Foundation (grant No. 81804153), Guangzhou Science and Technology Plan Foundation and Application Foundation Research Project (grant No. 202102020020), Guangdong Foundation for Basic and Applied Basic Research (grant No. 2019A1515110161), Fundamental Research for the Central Universities (grant No. 21621410), Scientific Research Project of Guangdong Provincial Administration of Traditional Chinese Medicine (grant No. 20201107), and Starting fund for doctoral research of the Sixth Affiliated Hospital of Jinan University (grant No. JDLY2022001).

## Conflict of Interest

The authors declare that the research was conducted in the absence of any commercial or financial relationships that could be construed as a potential conflict of interest.

## Publisher’s Note

All claims expressed in this article are solely those of the authors and do not necessarily represent those of their affiliated organizations, or those of the publisher, the editors and the reviewers. Any product that may be evaluated in this article, or claim that may be made by its manufacturer, is not guaranteed or endorsed by the publisher.

## Supplementary Material

Supplementary Figure S1 T test analysis of species differences at the phylum level between groups. **(A)** TC and H groups. **(B)** CP and H groups. **(C)** TC and CP groups. The left picture shows the difference in species abundance between groups, and each bar in the figure represents the mean value of species with significant differences in abundance between groups in each group.

Supplementary Figure S2 T test analysis of species differences at the genus level between groups. **(A)** TC and H groups. **(B)** CP and H groups. **(C)** TC and CP groups. The left picture shows the difference in species abundance between groups, and each bar in the figure represents the mean value of species with significant differences in abundance between groups in each group.

Supplementary Figure S3 T test analysis of species differences at the species level between groups. **(A)** TC and H groups. **(B)** CP and H groups. **(C)** TC and CP groups. The left picture shows the difference in species abundance between groups, and each bar in the figure represents the mean value of species with significant differences in abundance between groups in each group.

Supplementary Figure S4 PICRUSt predictions of the functional composition of the saliva microbiome. **(A)** KEGG pathway at level 1 between the TC and the H groups; **(B)** KEGG pathway at level 1 between the CP and the H groups; **(C)** KEGG pathway at level 2 between the TC and the H groups; **(D)** KEGG pathway at level 2 between the CP and the H groups. The left picture shows the difference in species abundance between groups, and each bar in the figure represents the mean value of species with significant differences in abundance between groups in each group.

Supplementary Figure S5 PICRUSt predictions of the functional composition of saliva microbiome at level 3 between TC and H group. The left picture shows the difference in species abundance between groups, and each bar in the figure represents the mean value of species with significant differences in abundance between groups in each group.

Supplementary Figure S6 PICRUSt predictions of the functional composition of saliva microbiome at level 3 between CP and H group. The left picture shows the difference in species abundance between groups, and each bar in the figure represents the mean value of species with significant differences in abundance between groups in each group.
