# Supplementary figures and images for: Salivary Microbiome Profile of Diabetes and Periodontitis in a Chinese Population
Source: Front Cell Infect Microbiol. 2022 Aug 1;12:933833. doi: 10.3389/fcimb.2022.933833 (PMC9377223; doi:10.3389/fcimb.2022.933833)

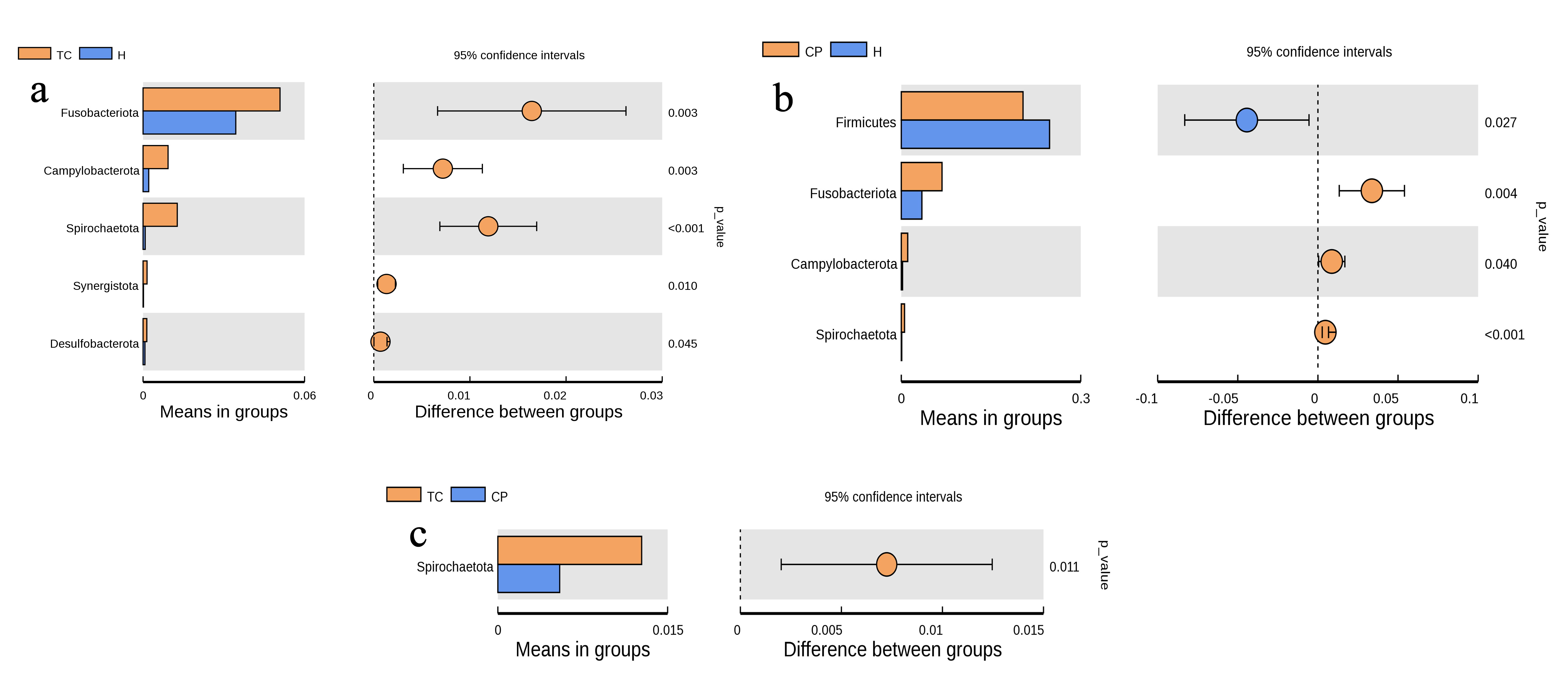

Supplement: Supplementary Figure S1 — T test analysis of species differences at the phylum level between groups. (A) TC and H groups. (B) CP and H groups. (C) TC and CP groups. The left picture shows the difference in species abundance between groups, and each bar in the figure represents the mean value of species with significant differences in abundance between groups in each group. [file Image_1.jpeg]

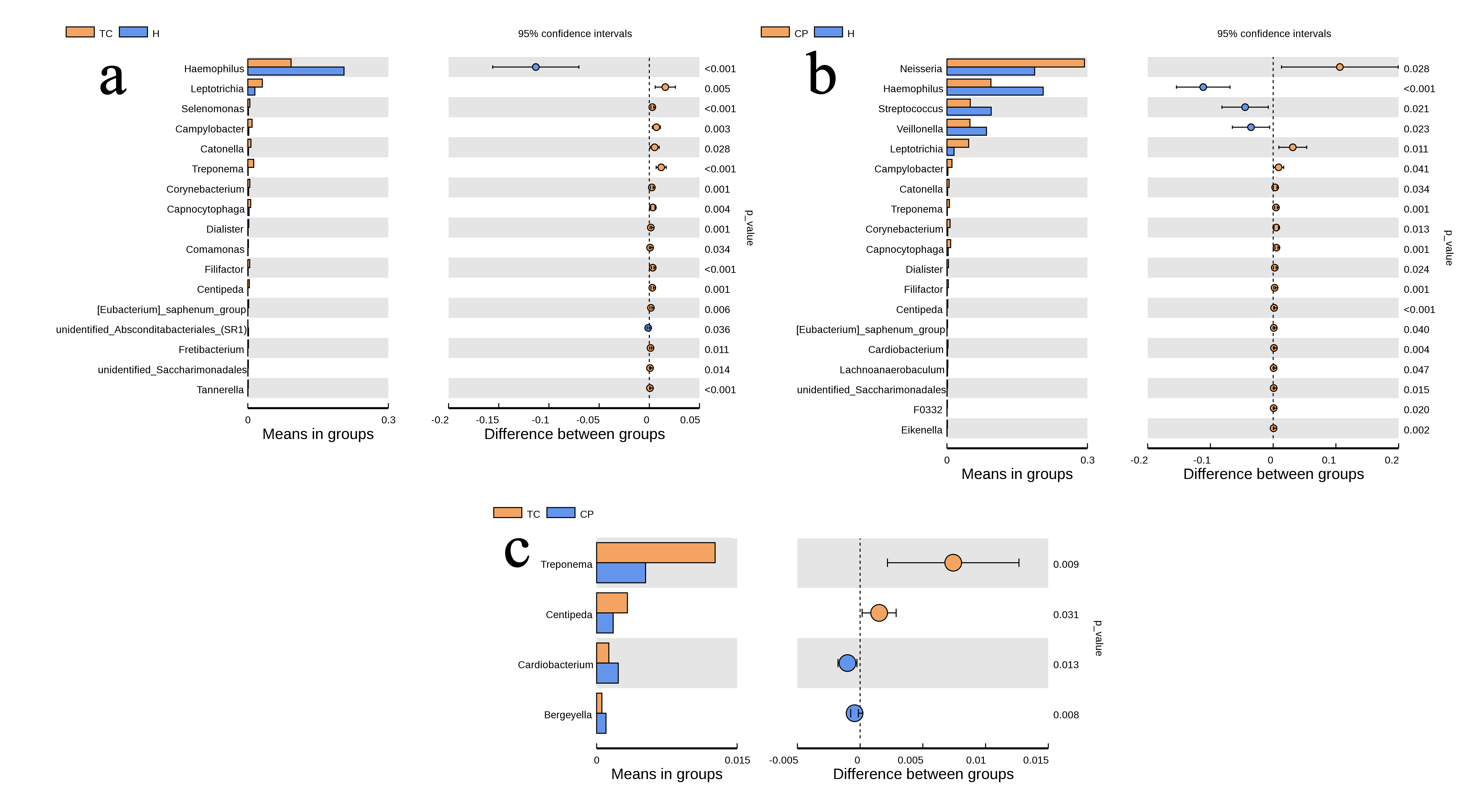

Supplement: Supplementary Figure S2 — T test analysis of species differences at the genus level between groups. (A) TC and H groups. (B) CP and H groups. (C) TC and CP groups. The left picture shows the difference in species abundance between groups, and each bar in the figure represents the mean value of species with significant differences in abundance between groups in each group. [file Image_2.jpeg]

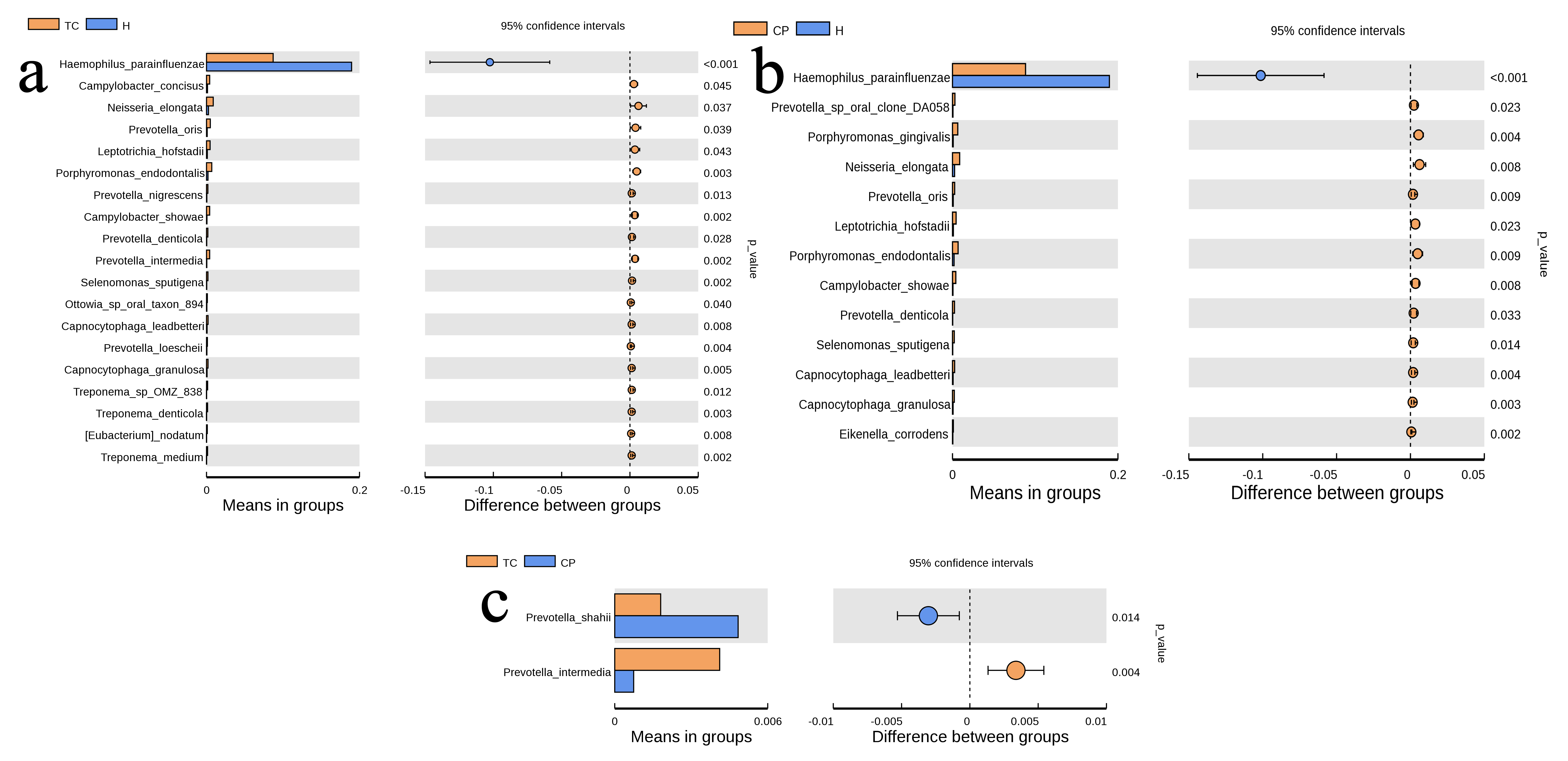

Supplement: Supplementary Figure S3 — T test analysis of species differences at the species level between groups. (A) TC and H groups. (B) CP and H groups. (C) TC and CP groups. The left picture shows the difference in species abundance between groups, and each bar in the figure represents the mean value of species with significant differences in abundance between groups in each group. [file Image_3.jpeg]

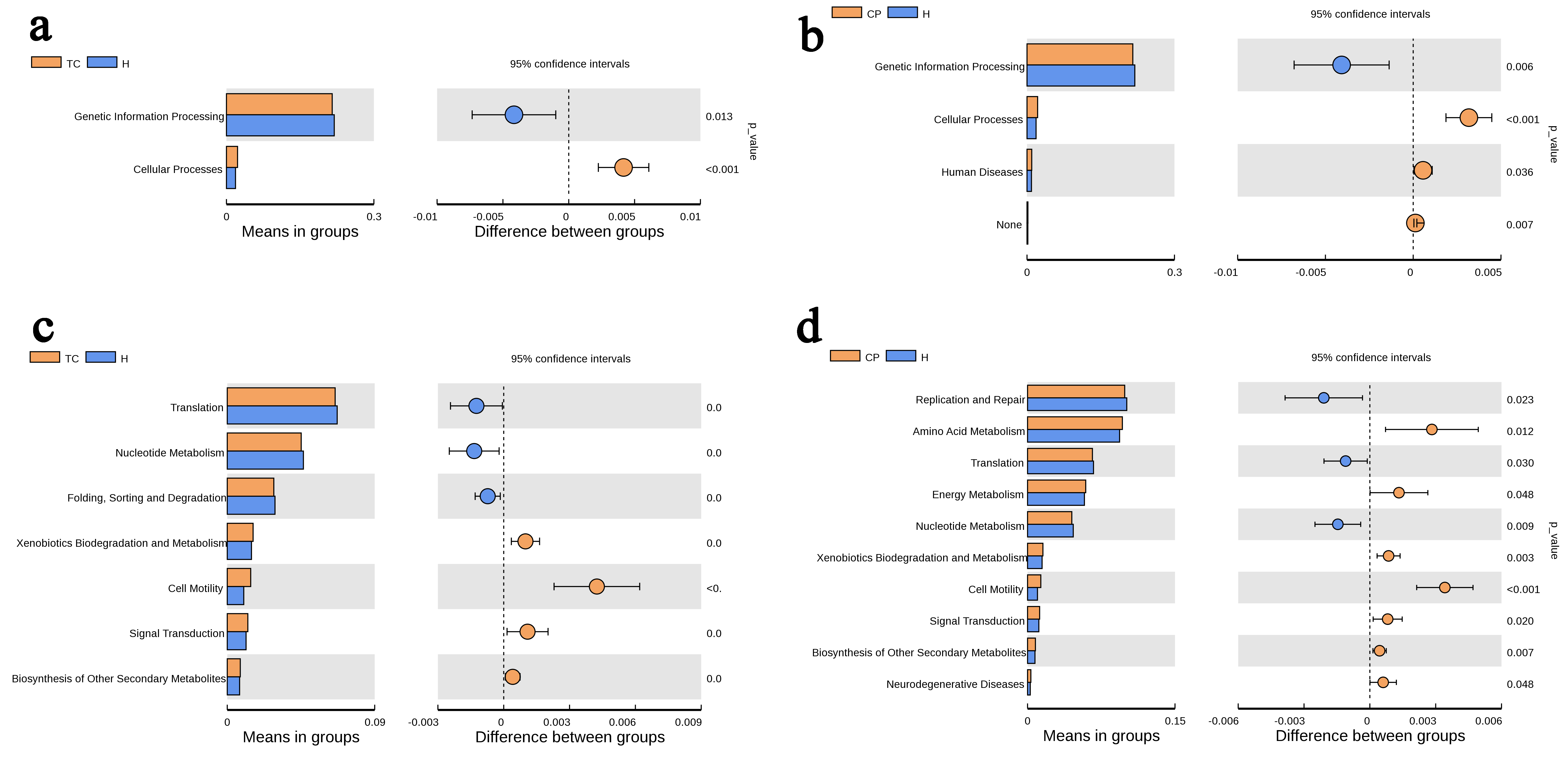

Supplement: Supplementary Figure S4 — PICRUSt predictions of the functional composition of the saliva microbiome. (A) KEGG pathway at level 1 between the TC and the H groups; (B) KEGG pathway at level 1 between the CP and the H groups; (C) KEGG pathway at level 2 between the TC and the H groups; (D) KEGG pathway at level 2 between the CP and the H groups. The left picture shows the difference in species abundance between groups, and each bar in the figure represents the mean value of species with significant differences in abundance between groups in each group. [file Image_4.jpeg]

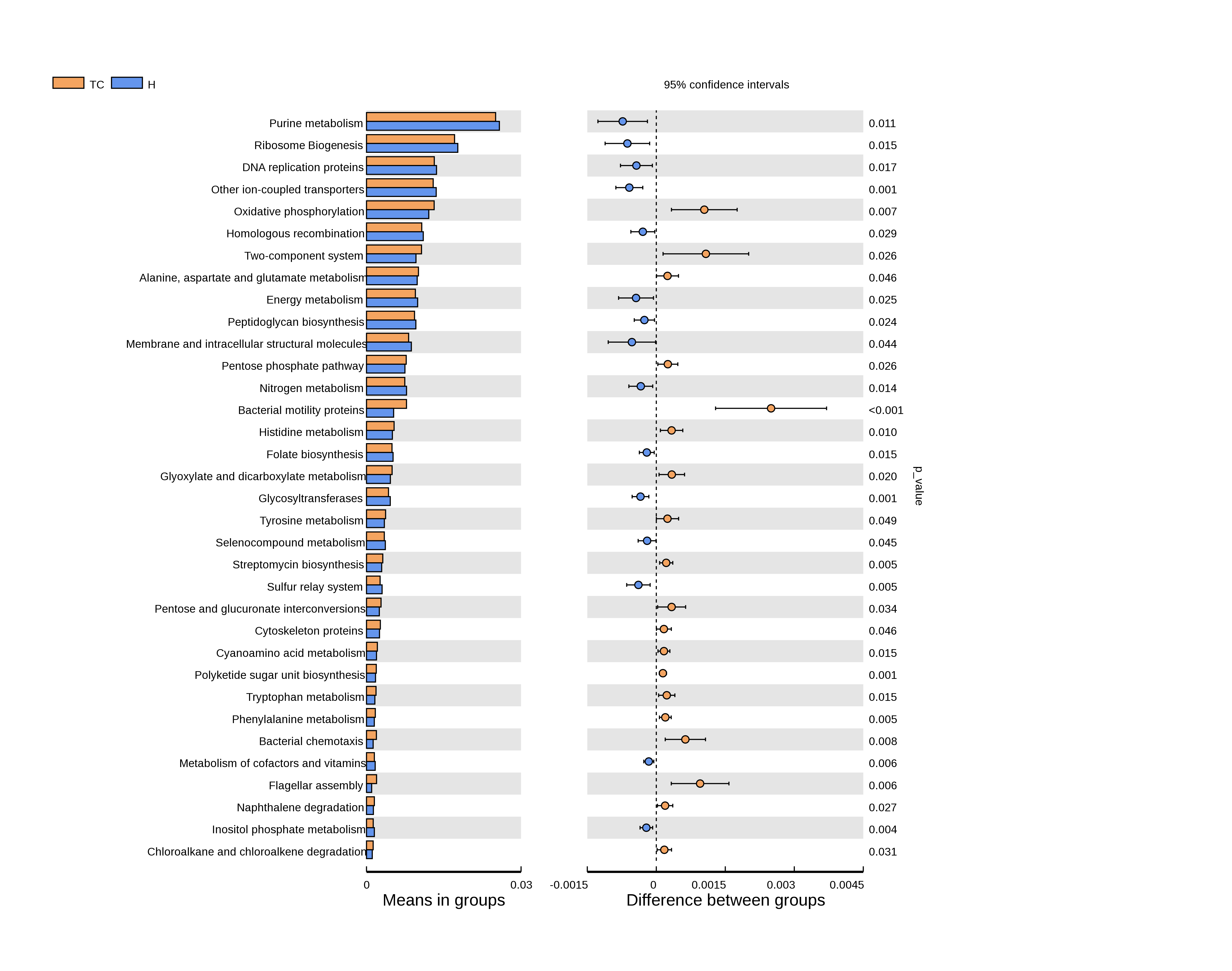

Supplement: Supplementary Figure S5 — PICRUSt predictions of the functional composition of saliva microbiome at level 3 between TC and H group. The left picture shows the difference in species abundance between groups, and each bar in the figure represents the mean value of species with significant differences in abundance between groups in each group. [file Image_5.jpeg]

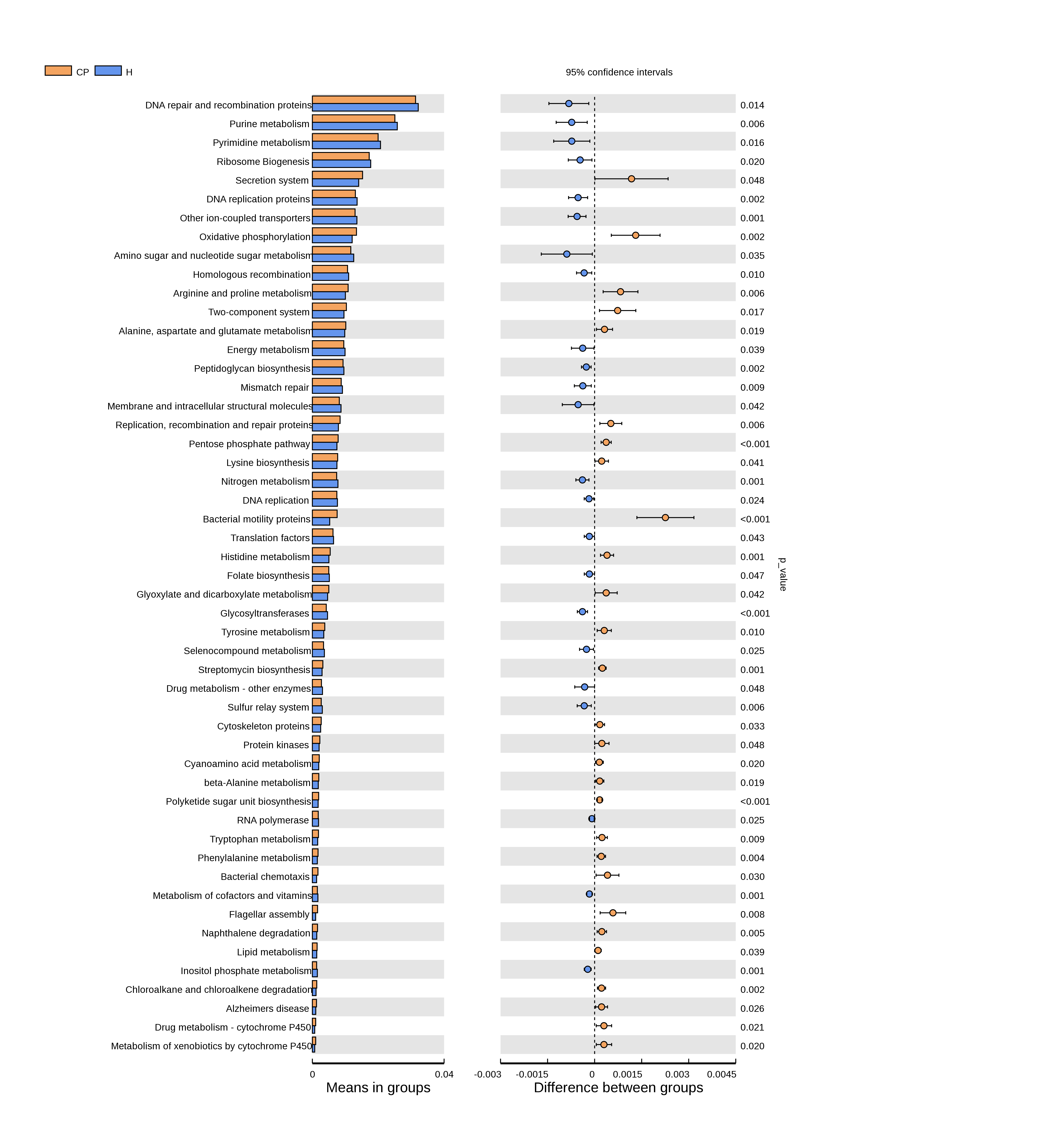

Supplement: Supplementary Figure S6 — PICRUSt predictions of the functional composition of saliva microbiome at level 3 between CP and H group. The left picture shows the difference in species abundance between groups, and each bar in the figure represents the mean value of species with significant differences in abundance between groups in each group. [file Image_6.jpeg]
